# Biomechanical Effects of Using a Passive Exoskeleton for the Upper Limb in Industrial Manufacturing Activities: A Pilot Study

**DOI:** 10.3390/s24051445

**Published:** 2024-02-23

**Authors:** Armando Coccia, Edda Maria Capodaglio, Federica Amitrano, Vittorio Gabba, Monica Panigazzi, Gaetano Pagano, Giovanni D’Addio

**Affiliations:** 1 Bioengineering Unit of Telese Terme Institute, Istituti Clinici Scientifici Maugeri IRCCS, 82037 Telese Terme, BN, Italy; armando.coccia@icsmaugeri.it (A.C.); gianni.daddio@icsmaugeri.it (G.D.); 2 Occupational Therapy and Ergonomics Unit of Pavia Institute, Istituti Clinici Scientifici Maugeri IRCCS, 27100 Pavia, PV, Italy; edda.capodaglio@icsmaugeri.it; 3 Department of Clinical-Surgical, Diagnostic and Pediatrics, University of Pavia, 27100 Pavia, PV, Italy; vittorio.gabba01@universitadipavia.it; 4 Occupational Therapy and Ergonomics Unit of Montescano Institute, Istituti Clinici Scientifici Maugeri IRCCS, 27040 Montescano, PV, Italy; monica.panigazzi@icsmaugeri.it; 5 Bioengineering Unit of Bari Institute, Istituti Clinici Scientifici Maugeri IRCCS, 70124 Bari, BA, Italy; gaetano.pagano@icsmaugeri.it

**Keywords:** wearable devices, inertial measurement units, electromiography, digital signal processing, occupational ergonomics, exposure assessment, upper limb repetitive actions, overhead work, arm-support exoskeleton, usability

## Abstract

This study investigates the biomechanical impact of a passive Arm-Support Exoskeleton (ASE) on workers in wool textile processing. Eight workers, equipped with surface electrodes for electromyography (EMG) recording, performed three industrial tasks, with and without the exoskeleton. All tasks were performed in an upright stance involving repetitive upper limbs actions and overhead work, each presenting different physical demands in terms of cycle duration, load handling and percentage of cycle time with shoulder flexion over 80°. The use of ASE consistently lowered muscle activity in the anterior and medial deltoid compared to the free condition (reduction in signal Root Mean Square (RMS) −21.6% and −13.6%, respectively), while no difference was found for the Erector Spinae Longissimus (ESL) muscle. All workers reported complete satisfaction with the ASE effectiveness as rated on Quebec User Evaluation of Satisfaction with Assistive Technology (QUEST), and 62% of the subjects rated the usability score as very high (>80 System Usability Scale (SUS)). The reduction in shoulder flexor muscle activity during the performance of industrial tasks is not correlated to the level of ergonomic risk involved. This preliminary study affirms the potential adoption of ASE as support for repetitive activities in wool textile processing, emphasizing its efficacy in reducing shoulder muscle activity. Positive worker acceptance and intention to use ASE supports its broader adoption as a preventive tool in the occupational sector.

## 1. Introduction

Exoskeletons (EXOs), designed to be worn on the body to support workers in physically demanding occupational settings, are suggested in certain work environments as a measure against fatigue and as a preventive intervention against risk factors associated with Work-related MusculoSkeletal Disorders (WMSDs). They are also considered a viable solution in cases where other preventive measures are impractical [1]. WMSDs, affecting 60% of European workers, exhibit prevalence rates of over 40% in upper limb-related disorders within the working population [2]. These disorders, caused by various factors, are particularly influenced or exacerbated by physical factors involving mechanical loads on musculoskeletal structures. Working conditions involving repetition, force, lifting, elevated upper limbs, or a combination of these factors for more than 1 h in a shift are associated with shoulder disorders [3,4], often categorized under the term ‘subacromial conflict syndrome’ [5]. Hand–arm elevation and shoulder load are correlated with a doubled risk of developing chronic shoulder disorders [6,7,8]. At the individual level, gender, age and body mass modulate the risk of exposure by interacting with physical occupational risk factors, together determining the likelihood of developing irreversible and long-term shoulder disorders [7,8]. Prompt preventive measures in the workplace can have a lasting impact on shoulder health, and, in this direction, the adoption of EXOs in industrial settings has gained momentum. However, its implementation is constrained by regulatory gaps, insufficient long-term efficacy evidence, and concerns about potential adverse events [9,10]. On the other hand, EXO design has improved, with lightweight, slim, and user-friendly devices now available on the market. Among the popular EXOs, passive Arm-Support Exoskeletons (ASEs) have demonstrated significant reductions (10–26%) in shoulder muscle activity during both field measurements and controlled laboratory studies [1,11,12,13,14]. Field studies so far conducted in automotive, manufacturing, logistics, and agriculture sectors are essential to assess the effectiveness, appropriateness, safety, and user acceptance of occupational EXOs [15,16,17,18,19].

Exoskeleton studies have not yet considered the industrial textile sector, which involves manual interventions with high-frequency repetitive activities, weight handling, and incongruous upper limb postures. This is determined by the type of production machinery (spinning machines, winders and twisters) with work positions densely arranged along the longitudinal dimension of the machinery, and manual interventions performed on the machinery within a range of variable heights (from 70 cm to 210 cm). The worker’s activity mainly involves an upright position, frequent ground-walking alongside the machine, and continuous and repetitive movements of the upper limbs. These movements often require shoulder flexion beyond 80° and involve pinching or grasping threads or spools at different heights of the machine. Employees on shift can be assigned alternating periods of time on different machines, depending on organizational needs. This can result in variable biomechanical risk exposure. Preventive measures in this complex environment focus on risk containment, which is only partially addressed by organizational measures such as task rotation and duration reduction. In this context, ASE appears to be a cost-effective solution applicable to all workers.

This pilot study aims to assess the effectiveness of a commercially available passive ASE during typical tasks in the industrial processing of woolen textiles, based on the analysis of objective metrics derived from biomedical signals estimating the muscle fatigue and subjective feedback given by workers. This information can be used to plan interventions at the company to promote the use of exoskeletons. It can also be used to set up long-term monitoring of the various effects associated with the use of these devices.

The study employed a compact system to analyze surface electromyography signals, which is ideal for field studies where real movements can be investigated. This ensures greater relevance compared to laboratory studies, where researchers can only attempt to reproduce the actual conditions being analyzed. The research activity in the field of composite and hybrid materials is driving the development of new wearable sensors [20,21]. These sensors are small and can be in contact with the subject’s body, providing accurate and realistic measurements. In recent years, there has been a rise in proposed solutions in textile technology for medical purposes, particularly for diagnosis and monitoring outside of the laboratory [22]. This increase is not coincidental.

The next chapter will describe in detail the materials and methods used in this work. In particular, the exoskeleton used during the experimental tests and the study design will be described, with reference to the experimental procedure, the muscle activity parameters and the usability and user satisfaction questionnaires, as well as the statistical methods used for the analysis. The results are presented in Section 4, while their interpretation is provided in the discussion section. The analysis of the effect of the exoskeleton on muscular activity is supplemented by a correlation analysis between the variables collected in the study, which is treated separately for the sake of clarity. Finally, concise conclusions deduced from the statistical analysis are reported.

## 2. Materials and Methods

**Figure 1:**
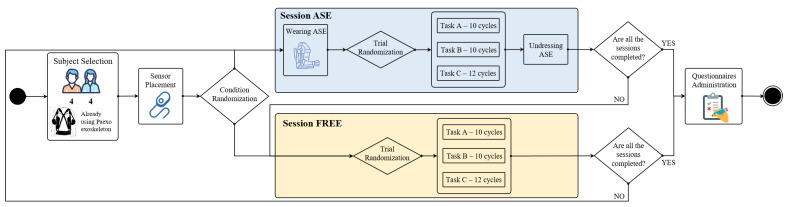
Flowchart describing the experimental procedure.

Figure 1 provide a graphical representation of the experimental procedure described in the following paragraphs.

### 2.1. Study Population

Our pilot study was conducted in Northern Italy at a textile company as part of an ongoing experiment, where the employer provided a novel passive ASE, for voluntary use by employees during specific work tasks. Among these workers, we selected a small sample for our study. In recruitment, we ensured an equitable distribution of gender and age among workers to increase the representativeness of the sample with respect to the real working population. The sample comprised eight participants, with an equal gender split of four men and four women. Table 1 provides detailed demographic characteristics of the participants. The only criteria for exclusion from participation in the study were the presence of ongoing acute disabling conditions that do not allow the performance of repetitive activities with the upper extremities, or the presence of internal complications that contraindicate the performance of activities involving the lumbopelvic spine.

This research project underwent ethical review beforehand and received official approval from the Ethics Committee, identified by the approval number 2732 EC. Each participant received detailed information about the research’s objectives and methods and voluntarily confirmed their participation in the study.

### 2.2. Exoskeleton

The passive upper limb EXO Paexo Shoulder (Ottobock, Duderstadt, Germany) was tested in the experimental study. This specific type of EXO appears in the literature to have already undergone a process of evaluation (assessment in work-like tasks) but not validation (assessment in a controlled laboratory setting with no particular connection to the work task) nor field assessment in the sense of real-world assessment [11].

**Figure 2:**
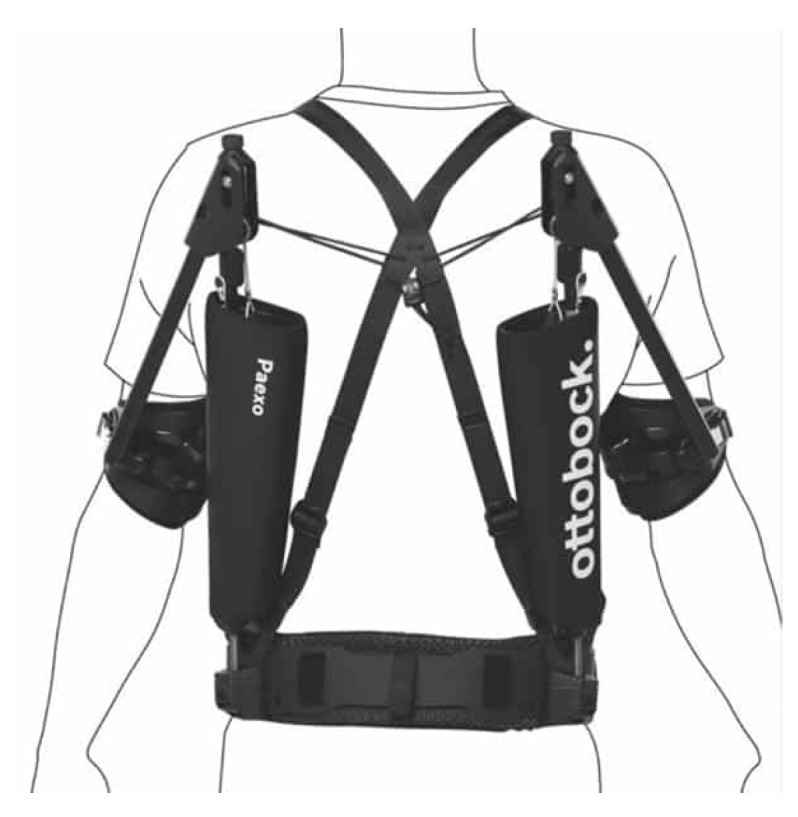
Paexo Shoulder (Ottobock, Duderstadt, Germany), worn by a participant of the study in rest position before carrying out the tests.

The Paexo Shoulder EXO (2019) consists of a lightweight frame (total weight 1.99 kg) in the form of a backpack worn on the back of the trunk (Figure 2). The design includes supports attached to the arms, leaving free the movement of the trunk and upper extremities. The support torque varies with the arm elevation angle, reaching its maximum at an elevation angle of 90° (upper arm horizontal), and becomes zero when the arm is lowered along the body. The primary goal of these assistive EXOs is the reduction in effort in the upper limbs.

### 2.3. Experimental Procedure

**Figure 3:**
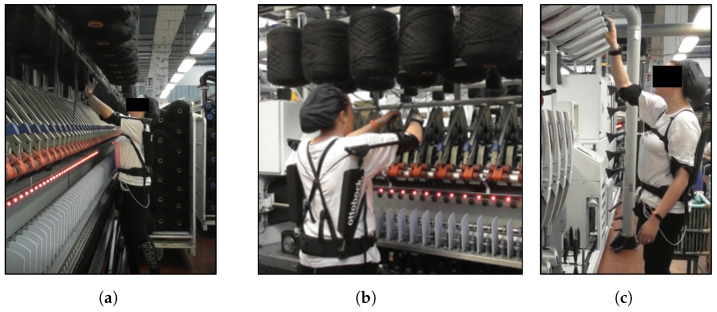
A participant performing the activities using the exoskeleton. (**a**) In Task A, the worker lifts and fix the spool to a pin on the higher part of the machinery, (**b**) in Task B, the worker takes a couple of threads and inserts them into the lower part of the machinery operating a lever and (**c**) in Task C, the worker places a dozen tubes to the top of the twisting machine.

The experimental protocol comprised two consecutive sessions for each participant: one involving the use of the ASE and the other without the support (FREE). The order of these sessions was randomized to mitigate the potential order effects in statistical analysis, and each participant acted as their own control, as the intra-individual differences in the variables of interest between the two conditions were considered in the analysis. Each session included three types of dynamic repetitive tasks commonly performed by workers on machinery, specifically twisting and winding machines, which predominantly involve overhead work (Figure 3).

During the experimental sessions, the pace of task execution (time per cycle) was not strictly fixed. The worker was instructed to complete the required number of cycles but was free to adopt their usual work pace and preferred technique. The experimentation was conducted on standard machinery, resulting in the same geometric working points for all subjects. For individuals who are short in stature, reaching the highest points of the machine requires greater postural effort on the shoulders.

Activity A involves the worker lifting a spool (≈3–3.5 kg) from a service trolley with both hands and raising it to a height of 200 cm by applying pressure to fix it onto a pin (as shown in Figure 3a). The single cycle was made up of two spools fixed on two adjacent pins (either front or rear row), and was repeated 10 times in a session, with an average duration of 80 s. The shoulders are flexed more than 80° for 25% of the cycle.

Task B involves operating the spinning machine pre-loaded with five pairs of spools (anterior and posterior row). The worker is required to take a couple of threads, slip them with both hands from a spool at a height of 200 cm, and insert them into the lower parts of the machine. During 55% of the cycle, the shoulders are flexed >80° (refer to Figure 3b). Additionally, in each cycle, the right hand operates a lever, applying a medium-light force (≈2.5 kg). Each cycle involves positioning two threads for one spool and lasts approximately 11 s. This process is repeated 10 times throughout the task.

In task C, the worker transfers a set of dozen tubes (<100 g each) from a trolley to the top of a twisting machine at a height of 210 cm. Typically, the worker holds the tubes with his left hand and uses his right hand for insertion (Figure 3c). Each cycle (one tube) lasts 2 s, for a total of 12 recorded cycles.

These three tasks vary in physical demands, including cycle duration, load handling and percentage of cycle time with shoulders flexed (>80°) (Table 2). These differences in physical exertion were reflected in the different risk indices for each task, calculated using the OCcupational Repetitive Actions (OCRA) method, developed by Occhipinti et al. [23]. The OCRA index categorizes risk levels into at least three classifications: no risk, uncertain or very slight risk, and the presence of risk. This categorization is grounded in predicting the likelihood of injury due to exposure levels. The OCRA method entails a comprehensive analysis of the work task, assigning scores to various risk factors, such as repetitiveness, duration, posture, exertion, recovery periods, and complementary factors. Based on these factors, Task A showed an OCRA index of 3.4 for the left side and 8.7 for the right side, placing it in the yellow (borderline) and red (moderate risk) zones, respectively. The OCRA index for Task B (threading multiple threads) was 2.1 for the left side and 2.3 for the right side, resulting in an acceptable risk window (green zone) and a borderline risk window (yellow). Finally, Task C (tube replacement) had a OCRA index of 1.1 for the left side and 14.0 for the right side, corresponding to a green (acceptable) and purple (high risk) risk window, respectively. In summary, the dominant (right) side was at borderline-to-high risk in tasks A, B and C.

### 2.4. EMG Feature Extraction

**Figure 4:**
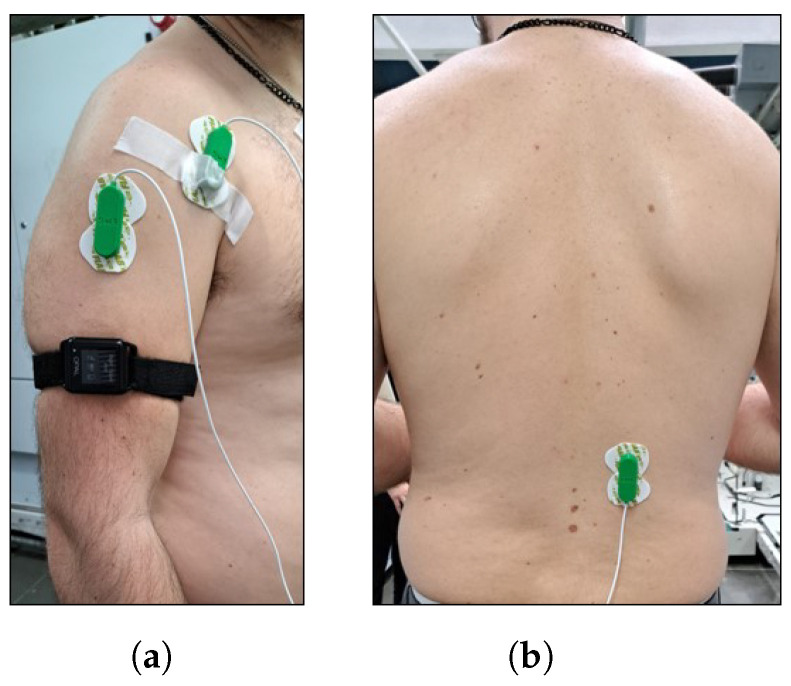
Electrode placement on the anterior and medial deltoid muscles (**a**) and the ESL (**b**), following SENIAM guidelines. This standardized positioning facilitates reliable EMG signal acquisition for the assessment of dynamic upper limb and back muscle activities.

At the start of each session, participants were equipped with adhesive surface electrodes connected to wearable systems to detect electromyography (EMG) activity. EMG data were collected using the assembled EMG Sensor BITalino (r)evolution BLE system (gain 1009, input impedance 9.5 GOhm, CMRR 86 dB and sampling frequency 100 Hz), designed for real-time physiological data recording, coupled with the OpenSignals (r)evolution software (Public Build 2022-05-16; PLUX Wireless Biosignals S.A., Lisbon, Portugal) Three muscles of particular interest were studied: the right anterior deltoid, medial deltoid (Figure 4a), and the right Erector Spinae Longissimus (ESL) (Figure 4b). Each plays a crucial role in many human motor activities, also in the tasks examined in this protocol. Right-side muscles were monitored since participants were all right-handed. Electrodes were placed on these muscles, following Surface Electromyography for the Non-Invasive Assessment of Muscles (SENIAM) recommendations [24] (Figure 4). EMG signals were processed in MATLAB R2023a (The Mathworks, Inc., Natick, MA, USA) to extract quantitative metrics estimating muscle fatigue exerted during the tasks.

Signals were first manually segmented to identify the single cycles composing the session, and in each window, the Root Mean Square (RMS) and the Peak-to-Peak (P2P) amplitude were extracted from the signal. These parameters represent quantitative measures useful for characterizing the signal, and therefore, the activity, of the muscles analyzed. Although they cannot be considered an absolute measure of muscle fatigue when not normalized, in this study, the analysis was conducted on intra-subject variations due to the use of the exoskeleton. The use of statistical tests for paired data only takes this variable condition into account, as all other factors are fixed in the experimental setting.

### 2.5. Usability and Satisfaction Assessment

To assess the user satisfaction and usability of the EXO used in the study, two different but complementary instruments were submitted to the study population: Quebec User Evaluation of Satisfaction with Assistive Technology (QUEST) and System Usability Scale (SUS). The QUEST, developed by Demers et al. [25], served as the tool for assessing workers’ satisfaction with both technical and usability aspects of the EXO, as well as their contentment with the delivery and support they received. We analyzed the responses by categorizing them into two groups based on their satisfaction levels: low satisfaction (scores ranging from 1 to 3, representing “not at all satisfied” to “moderately satisfied”) and high satisfaction (scores between 4 and 5, denoting “satisfied” to “very satisfied”).

This categorization enabled a clear distinction of participants’ satisfaction levels with the EXO. The QUEST framework operates on the premise that user satisfaction with assistive technology is shaped by personal expectations, perceptions, attitudes, and evaluations. It places greater emphasis on specific features and associated services rather than solely focusing on equipment performance.

The SUS, as elucidated in [26], functioned as a tool for evaluating the usability of technology. This scale entails user ratings on 10 constructs, expressed through statements concerning product usability, which are assessed on a Likert scale ranging from 1 (“strongly disagree”) to 5 (“strongly agree”). The final score, calculated as the sum of individual responses, yields a comprehensive measure of the overall usability of the system. The underlying principle of SUS is that usability is contingent on context, closely intertwined with the environment, the activity conducted, and the user. It encompasses three pivotal aspects: efficiency, effectiveness, and user satisfaction.

### 2.6. Statistical Analysis

#### 2.6.1. Two-Way ANOVA

To evaluate differences in muscle activation among subjects in the ASE and FREE conditions during the performance of the previously described tasks, a two-way analysis of variance (Two-Way ANOVA) was employed on three distinct datasets, each corresponding to a different muscle district (anterior deltoid, medial deltoid, and ESL).

The two-way ANOVA enables the simultaneous assessment of two categorical variables’ impact on a continuous quantitative variable. Its advantage over the one-way ANOVA lies in testing the relationship between two variables (factor and dependent variable), while considering the influence of a third variable (another factor). In this instance, the two qualitative variables are ’subject’ and ’condition’ (task performed with the EXO vs. without the EXO). The quantitative dependent variables include parameters derived from the EMG signals of each muscle.

The results of this analysis can highlight statistically significant effects of the EXO on muscle effort, accounting for inter-subject differences. The significance level was set at 0.05.

#### 2.6.2. Correlation Analysis

Spearman correlation was used to assess the association between the effects of the EXO and demographic variables of the population. The effects of the EXO on muscle effort were quantified by calculating the difference between the RMS values assessed in the two working conditions (ΔRMS). Positive values of ΔRMS indicate reduced muscle effort when using the EXO, while negative values indicate higher EMG signal RMS values when the support is worn by the worker. Spearman correlation is a non-parametric statistical technique used to determine the existence and direction of a monotonic relationship between ordinal or continuous variables that may not adhere to a normal distribution.

The Spearman correlation coefficient, denoted as ρ, ranges from −1 to 1. A ρ value near +1 signifies a strong positive monotonic correlation, while a value near −1 indicates a strong negative monotonic correlation. A value near 0 suggests no monotonic correlation between the variables. All statistical analyses were carried out using Jamovi software ver. 2.3.28 (Jamovi Project, Sydney, Australia).

## 3. Results

### 3.1. Analysis of Exoskeleton Effect

The first analysis aimed to verify the impact of the use of the EXO in reducing muscular effort by means of the two-way ANOVA statistical technique run on the features extracted from the EMG signals.

**Figure 5:**
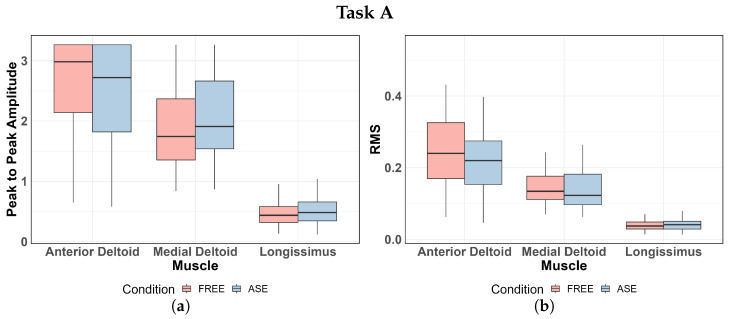

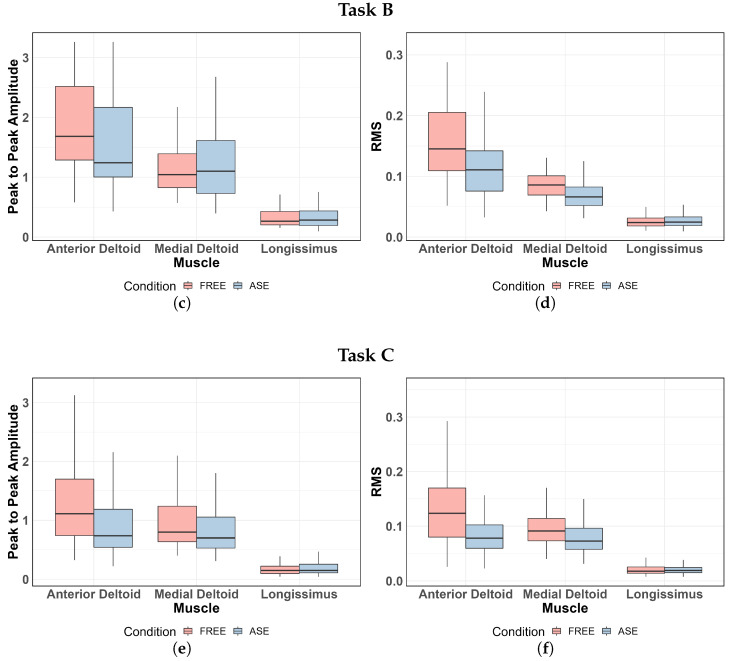
Distribution of EMG P2P amplitude (**left**) and RMS (**right**) in the muscles analyzed: (**a**,**b**) Task A; (**c**,**d**) Task B; (**e**,**f**) Task C.

The boxplots in Figure 5a,b illustrate the distribution of EMG parameters in Task A. Descriptive statistics (mean ± std) and the results of the ANOVA (*p*-value) are shown in Table 3. Visual analysis of the boxplots indicates an average reduction in P2P amplitude and RMS of the anterior deltoid EMG signal when the EXO is utilized. A similar effect is observed in the medial deltoid muscle for the RMS parameter. These observations are substantiated by the statistical analysis: the ANOVA test highlights statistically significant differences in both parameters for the anterior deltoid and in the RMS value for the medial deltoid. Further significance is found in the differences in P2P amplitude for the ESL muscle. However, in this case, the values are increased in tasks where the EXO is used.

For Task B, the distribution of the EMG parameters is reported in Figure 5c,d. Table 4 shows descriptive statistics and ANOVA results in terms of the *p*-value of the factor condition of use (ASE vs. FREE). Statistical analysis confirms the positive impact of the EXO in reducing the P2P amplitude and RMS of the EMG signal of the muscle anterior deltoid and RMS in the medial deltoid also in Task B, while statistically significant effects are not registered for the muscle ESL activity. The reduction in RMS of the signal for the two shoulder muscles are relevant (*p*-value <0.001), higher than those shown in Task A.

In Task C, the impact of EXO use is similar to that of the other work tasks. The statistical significance of the difference between the conditions of use of the ASE is underlined for the anterior deltoid in P2P amplitude and RMS, and for the RMS of the signal recorded on the medial deltoid. The distribution of the values, reported in the boxplots of Figure 5e,f, shows the reduction in EMG features when the EXO is worn by workers. Table 5 shows the statistical descriptors of the distribution and the significance level of the ANOVA test.

### 3.2. Assessment of Satisfaction and Usability

**Figure 6:**
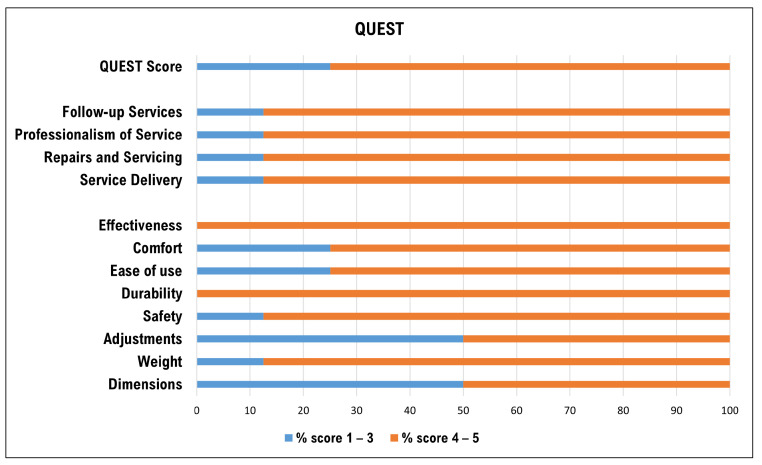
Satisfaction responses in relation to the delivery service (top) and in relation to the EXO (bottom). The abscissa shows the percentage of subjects declaring themselves to be slightly (in blue) and very satisfied (in orange), respectively.

The evaluation of user satisfaction and usability of the EXO was investigated using two scales: QUEST and SUS. QUEST overall scores, assessed by participants on a Likert scale (from 1 to 5, where 1 is “not satisfied at all” and 5 is “very satisfied”), were divided into two groups to identify a low satisfaction level (scores from 1 to 3) and a high satisfaction level (scores from 4 to 5). Regarding the device as shown in Figure 6, 100% of participants were completely satisfied with the durability (mean 4.37; std ±0.52) and effectiveness (mean 4.75; std ±0.46). The weight (mean 4; std ±0.93) and safety (mean 4.12; std ±0.64) of the device were fully satisfactory for 87.5% of the participants, while ease of use (mean 4.25; std ±1.16) and comfort (mean 3.62; std ±0.74) were satisfactory for 75% of the workers. The lowest levels of satisfaction were observed for adjustability (mean 3.5; std ±0.93) and dimensions (mean 3.5; std ±0.53). Satisfaction with the supply service (all items) reached full agreement for 87.5% of workers: delivery (mean 4.25; std ±0.71), assistance (mean 4.37; std ±0.74), professionalism (mean 4.12; std ±0.64) and follow-up service (mean 4.25; std ±0.71).

**Figure 7:**
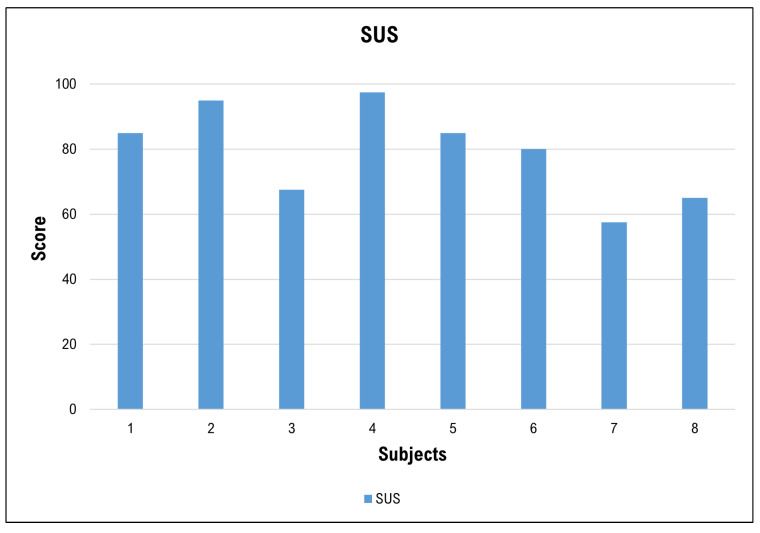
For each worker interviewed (in abscissa), the score obtained on the SUS usability questionnaire is shown (max score 100).

Figure 7 shows the SUS scores assigned by the workers. The highest score was 97.5 (out of a maximum score of 100), and the lowest was 57.5 (average of 79.06, std ±14.45). In percentile terms, 87.5 is at the 75th percentile, 82.5 at the 50th percentile, and 66.9 at the 25th percentile. Sixty-two percent of the scores are above the value of 80 (5 out of 8 subjects), indicating a predominantly good perceived usability of the EXO.

### 3.3. Correlation Analysis

Based on the previously presented findings regarding the impact of EXO usage on surface EMG parameters, it can be inferred that the parameter most indicative of the aid’s benefits is the RMS, which serves as a representative measure of the signal’s ‘power’. Consequently, we opted to include in the correlation analysis the difference (Δ) between the RMS values observed in trials without the EXO and those calculated from signals acquired during EXO use. Positive ΔRMS values indicate a reduction in muscle power during the task with the EXO, while negative values suggest the opposite effect.

The correlation analysis involved examining the ΔRMS value for each muscle in every task and for each subject in relation to various subject characteristics. These characteristics encompassed demographic variables such as age, Body Mass Index (BMI), length of service, and months of EXO use. Additionally, the analysis considered variables from the QUEST aid satisfaction questionnaire (including its individual components), usability questionnaire (SUS), and biomechanical risk classification (OCRA).

#### 3.3.1. Individual Variables

Table 6 shows the correlation coefficients determined between the sample of data describing the effects of the EXO on muscle activity (ΔRMS) and the individual variables. Absolute values of correlation higher than 0.7 are marked with an asterisk.

**Figure 8:**
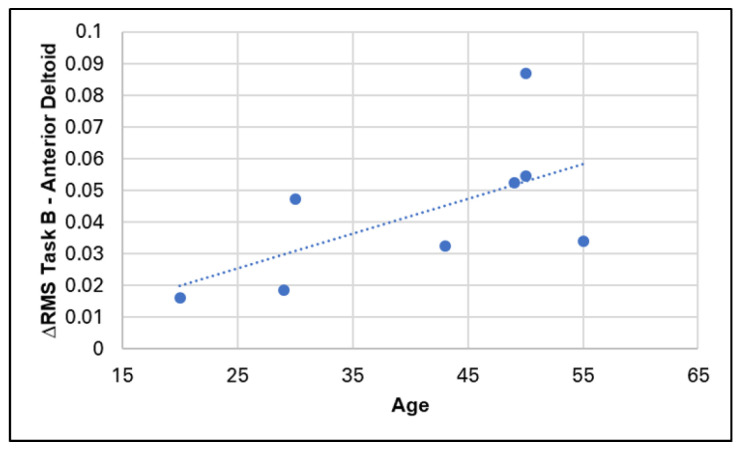
Distribution of age values and effect of the EXO on the anterior deltoid muscle in task B, for which the correlation is significant.

The only correlation value surpassing the defined threshold of 0.7 is the one linking the subject’s age to the reduction in effort of the anterior deltoid muscle in Task B (Figure 8). The other correlation indices fall below this threshold and are therefore not considered significant within the exploratory nature of the analysis.

#### 3.3.2. OCRA Classification

In conclusion, we examine the correlation between the effects of the EXO on the utilized muscle strength (measured by the ΔRMS parameter) and the OCRA classification across three distinct tasks. The inquiry aims to ascertain whether a relationship exists between the EXO’s effects and the biomechanical risk associated with each task. Notably, Spearman’s correlation coefficient cannot be employed for this analysis, given that the OCRA classification is task-specific and not relative to individual subjects.

**Figure 9:**
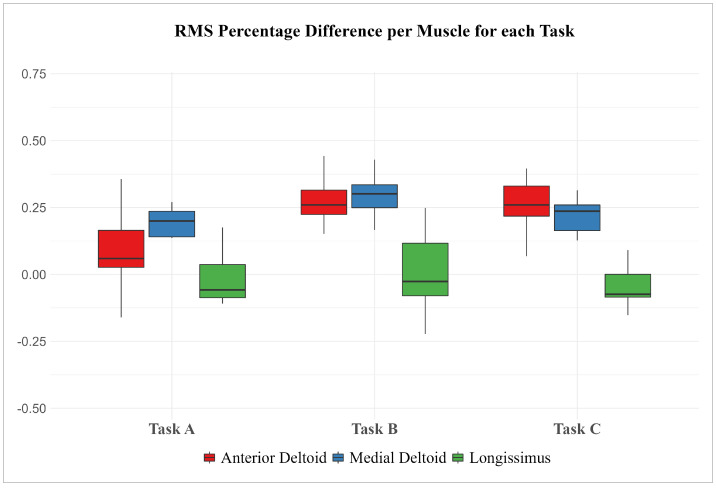
Representative boxplots of ΔRMS values for each muscle, divided by Task.

Table 7 displays the OCRA classification values for each task, exclusively for the right limb, where electromyographic data were recorded. Additionally, Figure 9 presents representative boxplots illustrating the ΔRMS, in percentage values, categorized by muscle and task.

## 4. Discussions

As suggested in the literature, even though there is no standardized procedure for assessing the effectiveness of EXOs, it is important to understand the evaluative metrics, both objective (e.g., surface electrodes and motion sensors) and subjective (user’s perception and feedback regarding the EXO). This understanding should be contextualized with respect to the type of posture and tasks analyzed [27].

In our study, we considered the comparison between the conditions of EXO use (ASE) and non-use of the EXO (FREE) during the performance of three industrial tasks involving repetitive upper limb activities and overhead work, carried out by industry workers. These tasks were selected, as they are routinely performed by workers during their shifts and involve repetitive activities and overhead work, factors associated with shoulder disorders.

The participants in the study were volunteers selected from a sample of workers in the sector who had previously agreed to the employer’s proposal to experiment with exoskeletons for a period of time, in order to test their usefulness in performing repetitive and overhead tasks. In selecting the workers, all of whom are experts in the sector, we took care to include an equal number of men and women and different age groups. This is in response to the criticism that in most of the published field studies on exoskeletons, the samples tested are not representative of the real working population [11].

The sample of real workers provides reliable results, and a fair representation of genders allows to consider aspects of usability and comfort related to the different body conformation of men and women. Furthermore, in the textile sector, the female gender predominates in the workforce due to greater manual dexterity in highly repetitive and precise activities. Gender, together with age and BMI, interacts with occupational risk factors to determine the likelihood of developing WMSDs, even in the long term [8].

Certainly, conducting tests in real-world conditions rather than in controlled laboratory environments may present constraints regarding the type of sensors used and the quality of signals. This is due to environmental and temporal factors, such as cluttered and noisy surroundings, the extent of workers’ movements, interferences, and limited time for equipment and calibration, as well as unforeseen events. In planning our field study at a textile production department with several noisy machines and a microclimate of around 26 °C and 70% humidity, the primary concern was obtaining “clean” signals from biometric data. In practice, it was found that EMG recording posed no issues.

### 4.1. Analysis of Exoskeleton Effect

The purpose of this analysis is to explore the differences in muscle activity during repetitive tasks under two conditions: using an ASE and without it (FREE). In this paragraph, we will explore the changes observed in the electromyographic parameters of specific muscles, namely the anterior deltoid, medial deltoid, and ESL. Statistical analysis has unveiled significant trends and patterns in muscle response, contingent on the condition. These findings offer valuable insights into the effectiveness and potential implications of incorporating EXOs in the work environment.

In the analysis of Task A, it is noteworthy that the parameters derived from the EMG signal of the anterior deltoid muscle exhibit a statistically significant reduction when the EXO is employed. Similar effects are observed in the lateral deltoid muscle; however, the differences between conditions are less pronounced. Notably, only the RMS shows a significant difference, implying that the condition influences the overall muscle signal strength but may not necessarily affect the P2P amplitude. Nevertheless, RMS is the metric most closely associated with muscle contraction force; thus, it can be argued that the use of the EXO reduces the force exerted by the shoulder region in carrying out the task of loading the spools.

Similar results emerge from the statistical analysis of EMG data recorded during the execution of Tasks B and C. In general, the anterior deltoid muscle is notably affected by the use of the EXO as highlighted by the statistically significant reduction in both characteristic parameters of the EMG signal. The activity of the lateral deltoid muscle also exhibits a statistically significant variation in the two usage conditions, with and without the EXO. However, as emphasized in Task A, there is a statistically significant reduction only in the RMS parameter and not in the P2P amplitude. On the contrary, the right ESL shows limited response to the use of the EXO, with no significant variations in EMG parameters observed between the two working conditions. Only in Task A, the P2P amplitude of the ESL muscle EMG signal is significantly increased, albeit with a small percentage variation. These observations find further support in the analysis of Figure 9, depicting the distribution of variations in the RMS parameter of the EMG signal with the use of the EXO. It is evident that the ΔRMS consistently averages above zero for both the anterior deltoid and medial deltoid muscles across all analyzed tasks, while the distribution centers around zero for the ESL muscle.

The study demonstrated a distinct effect in reducing the level of muscle activity in the two shoulder muscles by the EXO, while the impact on the ESL muscle is negligible as would be reasonably expected.

According to the recent review by Moeller (2022) [19], studies examining the effects of EXOs in occupational settings mostly focus on passive EXOs, primarily investigating muscle activity (shoulder, upper limb, and body) and secondarily exploring other kinematic, physiological, or usability parameters. According to the review, the Paexo device has been considered in three studies [28,29,30], which demonstrated (in lab assessment) its effectiveness in reducing shoulder muscle activity without negatively impacting trunk activity or compromising performance, eliciting favorable judgments from users.

The effects on the shoulder muscles are nevertheless appreciable, even when using other types of passive EXOs. Several studies in the literature demonstrate a reduction in the muscle activity of the anterior and medial deltoid during lifting tasks [31,32,33,34], working with the arms at the shoulder level [35,36], working overhead [35,36,37,38,39,40,41,42,43], or more specific tasks [35,42,44,45]. The evidence diminishes when analyzing the muscles of the back. Increased activity of the Iliocostalis lumborum muscle has been observed with the Fortis EXO during overhead work [37], and with the WADE EXO in lifting and maintaining the arms at shoulder level [33]. The latissimus dorsi muscle appears to be relieved of activity when using a passive EXO as reported in [30,35] in a study using the Paexo EXO. In our study, the actual reduction in shoulder muscles activity achieved through the use of the passive upper limb EXO aligns with the literature findings, as does the increase or the lack of evidence for variations in the right ESL muscle [46].

### 4.2. Correlation Analysis

The correlation analysis was conducted to understand whether there was a relationship between the extent of the benefits provided by the EXO in reducing muscular effort and individual variables.

It is essential to highlight that this analysis is characterized by a limited number of measurements (eight subjects), which restricts the specific robustness of the reported findings. Nevertheless, they provide a valuable initial indication, warranting further investigation.

Interestingly, there is no significant correlation between the effects of the EXO and the duration of device use (Table 6). This suggests that the benefits, or more generally, the effects of the EXO, remain independent of the frequency of use and are evident even in subjects using it for the first time. This does not exclude that longer familiarization periods with the device could have potential improved impact on individual behaviors [19]. Conversely, a significant positive correlation exists between the recorded benefits of the EXO on the anterior deltoid muscle in Task B and age (refer to Figure 8), indicating a more pronounced effect on older subjects. While no significant correlation is observed, it is noteworthy that Spearman’s indices tend to be predominantly negative when considering BMI. This implies that the impact of the EXO is potentially less significant as the body mass index increases. This aspect should be analyzed more thoroughly to ascertain which individual parameter potentially interacts with the degree of effectiveness of the EXO.

A noteworthy consideration relates to the exploration of the possible relationship between the effectiveness of the EXO in reducing muscular effort and the biomechanical load associated with the occupational task as assessed by the OCRA index. The three work tasks analyzed in our study have different characteristics in terms of physical demands but all involved repetitive work and overhead tasks. The diverse features of the three tasks led us to hypothesize a more pronounced effect of the exoskeleton in the more demanding task. However, this hypothesis was not supported by the analysis, which instead revealed a reduction in muscle activity (anterior and medial deltoid) in all three tasks in the ASE condition compared to the condition without the exoskeleton (FREE). In other words, the analyzed data do not provide evidence to suggest a more pronounced EXO effect for heavier tasks (Tasks A and C) compared to less biomechanically demanding tasks (Task B). This finding is significant, as it demonstrates the broad effectiveness of the exoskeleton in various types of repetitive and dynamic tasks characterized by “overhead work”.

### 4.3. Workers’ Opinions

The level of satisfaction expressed by the workers with the device (Figure 6) provides important clues about the type of exoskeleton and the salient features that are positively perceived by workers in the textile sector, and, along with the high usability score (Figure 7), indicate a favorable reception and more widespread use for the future. The perception of effectiveness and durability, together with the appreciation of the weight and safety of the device, are important prerequisites for a valid use of ASE in the analyzed production context and indicate that the type of ASE analyzed is suitable for the type of work gestures performed on textile machines. The lower levels of satisfaction expressed in relation to the comfort, size and adjustability of the device should guide the development of ASE models with improved ease of use.

Considering the Paexo, the perceived ease of use was generally good, particularly in relation to the simple design of the device, as well as its effectiveness and versatility.

The significant lightness of the design (1.9 kg) compared to the weight of other commercial ASEs (up to 5 kg), the absence of rigid structures that hinder trunk and upper limb movements and upper limb movements, and its slim structure combined with adjustable straps ensure good stability when used for different types of work activities.

According to the most recent review of field studies [10], usability was moderate to high for all types of EXO-ASE evaluated. However, the overall acceptance of exoskeletons in the occupational context is a complex phenomenon related to technology-induced self-efficacy beliefs, which in turn are modulated by the ability to reduce effort and the attributed utility of the device [47].

### 4.4. Significance of ASE as Preventive Intervention for WMSD

The literature still lacks evidence of a reduction in WMSDs associated with the use of ASE, although an actual reduction in muscle activity is considered beneficial and favorable in this regard. This gap should be filled by longitudinal or case-control studies, as currently only one author [48] reports evidence of a reduction in the need for medical care by workers using EXOs for long periods.

In general, published studies support the use of exoskeletons as a useful intervention for WMSDs control but also highlight the need to optimize the match between the device, activity, and user to maximize beneficial effects and minimize undesired outcomes [9].

Beyond the prevention of WMSDs in all workers, the adoption of exoskeletons can help ensure the continued employment of older individuals or those who have suffered injuries. Despite a reduction in their physical abilities, they can maintain satisfactory performance with the support of the device. This aspect is particularly relevant in the European and Italian work context and holds significant importance from a health, economic, and social perspective. However, as of now, no study has examined the effectiveness of exoskeletons in workers with musculoskeletal disorders in terms of actual work reintegration and the reduction in the risk of leaving employment or developing long-term disabilities [19].

## 5. Conclusions

The pilot study carried out on a numerically small sample of workers in the wool textile industry under real working conditions confirms the applicability in this context of the technique of measuring EMG signals using wearable sensors and demonstrates the effectiveness of the ASE in reducing shoulder muscles activity, regardless of the type of task performed. The type of ASE considered appears suitable for the work context and is judged positively by workers. These preliminary results may support further field studies aimed at obtaining more robust evidence about the effectiveness of ASE.

In the literature, there is still a lack of a comprehensive understanding of the impact of exoskeletons use on workers health, primarily due to the absence of long-term studies in real-world conditions [19], as well as methodological research limitations [46,49]. In general, study participants are young and novice subjects (i.e., not actual workers), and almost exclusively male. The numerical consistency of the considered samples is limited, preventing reaching the critical threshold of 80% power for differences. Other limitations include the consideration of specific indicators of workload and only body areas directly supported by the device. The time periods analyzed in the studies are quite short (from a few seconds to 45 min for simulated tasks and, at most, a work shift for direct field observations), making it impossible to extrapolate valid long-term conclusions.

Our study took place in real working conditions, including both male and female subjects of different age groups. The analyzed activities represented phases of cyclic tasks actually performed by workers during their shifts, with all participants being proficient in the technique. The short observation period we adopted (a few cycles of repetitive tasks) was chosen due to the pilot nature of the study, for organizational convenience (operations were conducted during actual production activities), and to avoid lengthy recordings while maintaining doubts about obtaining clean signals in a highly disturbed environment. Long-term effects resulting from the use of ASE cannot be deduced from our study.

According to the review by Baldassarre et al. (2022) [10], some authors report an increase in the perceived discomfort level by workers during exoskeleton use, especially in dynamic tasks. This effect is associated with friction, pressure, or thermal discomfort caused by the device under specific working conditions. Additionally, a higher level of discomfort is reported by female workers regarding the adaptation of the device due to anthropometric features. In our study, we did not specifically record the subjective perception of workers’ effort in the ASE condition compared to FREE, as the literature suggests that this indicator can be subject to a placebo effect [42] and does not reflect the actual bodily benefits that would become evident only after a period of at least 6 months [50]. Furthermore, the reduction in the perception of effort caused by ASE would be more impactful after performing static tasks compared to various dynamic tasks [44]. However, by itself, it does not affect the level of usability and the worker’s willingness to use the device unless accompanied by the perception of effectiveness [51].

Our field pilot study focuses on the industrial textile sector, which had not yet been considered in the literature on occupational exoskeletons. Interventions aimed at preventing WMSDs and maintaining employment for older workers or those with disabilities in work sectors with similar demands to the textile industry can benefit from the results obtained in our study, considering the demonstrated effectiveness in reducing muscular load on the shoulder area.

Regarding future research on exoskeletons applied in occupational contexts, firstly, randomized controlled studies are recommended [52], including prospective studies with numerically substantial samples [11], to provide robust evidence of efficacy for preventing WMSDs and to assess health and safety levels associated with device use [53]. Various body regions should be considered (including those not directly supported by exoskeleton) to exclude the onset of disorders or adverse events, and the studied samples should exhibit variability in terms of age, gender, and health status [46]. The potential unexpected effects resulting from exoskeleton use, related to mobility, postural control, and safety aspects [54] should be considered over a sufficiently long period. The adoption of standardized protocols [1,39] would facilitate comparisons between different models. In general, results from biomechanical analysis associated with the use of exoskeletons should contribute to advancing standards in occupational health and safety, promoting the development and implementation of specific tools for risk assessment associated with the use of such devices in work settings. The detection and monitoring of clinical and subjective aspects should proceed in tandem with the involvement of occupational physicians to establish targeted and effective adoption programs for exoskeletons in productive contexts, including identifying any unsuitable workers or tasks not suitable for their use [10].

## Figures and Tables

**Table 1:** Demographic characteristics of the study population.

	Age (Year)	Height (cm)	BMI	Exoskeleton Use (Months)
mean	40.8	172	23.1	4.29
std	12.7	7	2.9	5.87

**Table 2:** Task description.

Task	A	B	C
Single Cycle Duration (s)	8	10	2
Number of Cycles in the Task	10	10	12
Estimated time (s)	80	110	24
Technical Actions/min Left	30	36	7
Technical Actions/min Right	45	42	60
Shoulder Flexion > 80°, Dominant Side (% cycle)	25	55	80
Height Range of Hand’s Actions (cm)	50–200	115–210	110–208
Applied Force/Load per Cycle (kg)	3.5	2	-

**Table 3:** Descriptive statistics (mean ± std) and *p*-value resulting from ANOVA test of electromyographic parameters for the muscles involved in Task A.

	P2P Amplitude			RMS		
	FREE	ASE	*p*-Value	FREE	ASE	*p*-Value
Anterior Deltoid	2.60±0.84	2.46±0.88	**<0.001**	0.240±0.099	0.214±0.089	**<0.001**
Medial Deltoid	1.94±0.74	2.05±0.79	0.216	0.177±0.120	0.153±0.084	**<0.001**
ESL	0.472±0.202	0.526±0.255	**0.010**	0.039±0.015	0.041±0.017	0.226

*p*-values indicating statistically significant differences are highlighted in bold.

**Table 4:** Descriptive statistics (mean ± std) and *p*-value resulting from ANOVA test of electromyographic parameters for the muscles involved in Task B.

	P2P Amplitude			RMS		
	FREE	ASE	*p*-Value	FREE	ASE	*p*-Value
Anterior Deltoid	1.85±0.773	1.53±0.772	**0.001**	0.155±0.062	0.113±0.050	**<0.001**
Medial Deltoid	1.32±0.78	1.31±0.80	0.785	0.104±0.065	0.080±0.046	**<0.001**
ESL	0.322±0.158	0.329±0.157	0.532	0.026±0.011	0.157±0.011	0.979

*p*-values indicating statistically significant differences are highlighted in bold.

**Table 5:** Descriptive statistics (mean ± std) and *p*-value resulting from ANOVA test of electromyographic parameters for the muscles involved in Task C.

	P2P Amplitude			RMS		
	FREE	ASE	*p*-Value	FREE	ASE	*p*-Value
Anterior Deltoid	1.26±0.722	0.945±0.593	**0.001**	0.134±0.073	0.093±0.056	**<0.001**
Medial Deltoid	1.02±0.624	0.912±0.622	0.052	0.105±0.055	0.085±0.044	**<0.001**
ESL	0.170±0.094	0.184±0.108	0.052	0.020±0.009	0.021±0.009	0.152

*p*-values indicating statistically significant differences are highlighted in bold.

**Table 6:** Spearman correlation between the effects of the EXO on muscle activity (ΔRMS) and the demographic variables.

	Task A			Task B			Task C		
	Anterior Deltoid	Medial Deltoid	ESL	Anterior Deltoid	Medial Deltoid	ESL	Anterior Deltoid	Medial Deltoid	ESL
Age	0.419	0.0479	−0.299	0.707 *	−0.108	−0.491	0.275	0.204	−0.599
BMI	−0.405	−0.190	0.238	−0.619	−0.0476	0.0952	−0.381	−0.381	0.429
Length of Service	0.0120	1.59 × 10^−17^	−0.422	0.566	−0.193	−0.145	0.554	0.0482	−0.193
PAEXO Use	0.209	0.0491	−0.356	0.503	0.454	0.0614	0.381	0.221	−0.196

**Table 7:** OCRA task classification values, related only to the right limb on which the electromyographic data were recorded.

	Task A	Task B	Task C
OCRA Index	8.7	2.3	14.0

## Data Availability

Data are contained within the article.

## Abbreviations

The following abbreviations are used in this manuscript:

ASE	Arm-Support Exoskeleton
BMI	Body Mass Index
EMG	Electromyography
ESL	Erector Spinae Longissimus
EXO	Exoskeleton
OCRA	OCcupational Repetitive Actions
P2P	Peak-to-Peak
QUEST	Quebec User Evaluation of Satisfaction with Assistive Technology
RMS	Root Mean Square
SENIAM	Surface Electromyography for the Non-Invasive Assessment of Muscles
SUS	System Usability Scale
WMSD	Work-related MusculoSkeletal Disorder
